# Causal associations between sleep traits and brain structure: a bidirectional Mendelian randomization study

**DOI:** 10.1186/s12993-023-00220-z

**Published:** 2023-10-02

**Authors:** Qiao Wang, Shimin Hu, Lei Qi, Xiaopeng Wang, Guangyuan Jin, Di Wu, Yuke Wang, Liankun Ren

**Affiliations:** 1 Department of Neurology, Xuanwu Hospital, Capital Medical University, NO.45 Changchun Street, Xicheng District, Beijing, China; 2 National Center for Neurological Disorders, Beijing, China; 3 Beijing Key Laboratory of Neuromodulation, Beijing, China; 4 Institute of Sleep and Consciousness Disorders, Center of Epilepsy, Beijing Institute for Brain Disorders, Capital Medical University, Beijing, China

**Keywords:** Sleep hygiene, Brain structure, Mendelian randomization, Causal effect, Epidemiology

## Abstract

**Background:**

Emerging evidence suggests bidirectional causal relationships between sleep disturbance and psychiatric disorders, but the underlying mechanisms remain unclear. Understanding the bidirectional causality between sleep traits and brain imaging-derived phenotypes (IDPs) will help elucidate the mechanisms. Although previous studies have identified a range of structural differences in the brains of individuals with sleep disorders, it is still uncertain whether grey matter (GM) volume alterations precede or rather follow from the development of sleep disorders.

**Results:**

After Bonferroni correction, the forward MR analysis showed that insomnia complaint remained positively associated with the surface area (SA) of medial orbitofrontal cortex (β, 0.26; 95% CI, 0.15–0.37; *P* = 5.27 × 10^–6^). In the inverse MR analysis, higher global cortical SA predisposed individuals less prone to suffering insomnia complaint (OR, 0.89; 95%CI, 0.85–0.94; *P* = 1.51 × 10^–5^) and short sleep (≤ 6 h; OR, 0.98; 95%CI, 0.97–0.99; *P* = 1.51 × 10^–5^), while higher SA in posterior cingulate cortex resulted in a vulnerability to shorter sleep durations (β, − 0.09; 95%CI, − 0.13 to − 0.05; *P* = 1.21 × 10^–5^).

**Conclusions:**

Sleep habits not only result from but also contribute to alterations in brain structure, which may shed light on the possible mechanisms linking sleep behaviours with neuropsychiatric disorders, and offer new strategies for prevention and intervention in psychiatric disorders and sleep disturbance.

**Supplementary Information:**

The online version contains supplementary material available at 10.1186/s12993-023-00220-z.

## Background

Sleep, as a modifiable lifestyle habit, is essential for sustaining human life. It is characterized by multiple dimensions, including sleep quantity, quality, and circadian rhythm. Accumulating evidence suggests a complex bidirectional causality between unhealthy sleep patterns and neuropsychiatric disorders [1, 2]. Additionally, various sleep traits have emerged as potential markers or treatment targets for psychiatric disorders [3, 4]. For example, insomnia often precedes the onset of depressive disorder (DD) [5] and significantly increases the risk of developing DD in the future [6]; Sleep disturbances are prominent in patients with schizophrenia (SCZ) (up to 80% [7]), compared to the general population (around 20% [8]). However, the mechanisms linking sleep behaviours with neuropsychiatric disorders remain unclear.

Psychiatric disorders refer to a group of mental disorders characterized by psychological or behavioral abnormalities. Different brain structures have specific functions in controlling behavior and performance. Notably, the dorsolateral prefrontal cortex (PFC) and orbital frontal cortex (OFC) have been reported to be implicated in SCZ and bipolar disorder (BD), respectively [9]. Interestingly, these two brain regions have also been frequently identified as exhibiting morphological alterations in sleep-related MRI studies [10–16]. Considering that neuropathological changes often precede the clinical symptoms of neuropsychiatric disorders, it is hypothesized that variations in brain structure may underlie the causality between sleep habits and neuropsychiatric disorders, serving as either a cause or consequence of sleep patterns. Therefore, understanding the bidirectional causal relationships between sleep traits and brain imaging-derived phenotypes (IDPs) will help elucidate the mechanisms linking sleep behaviours with neuropsychiatric disorders.

At present, associations have been reported between global and regional differences in brain morphology and sleep quality in small case–control studies of insomnia, as well as moderate-sized observational studies conducted in general communities [10, 11, 15, 17–19]. However, the cross-sectional design of these neuroimaging studies limits their ability to infer the direction of the relationship between sleep patterns and brain structure. Furthermore, longitudinal studies addressing this issue have been unable to elucidate the temporal sequence between sleep habits and brain IDPs, as sleep conditions or brain magnetic resonance imaging (MRI) were evaluated at only one time point [16, 20–24]. Recently, the normal dynamics of bidirectional acute interaction between sleep duration and cortical thickness were revealed through microlongitudinal time series analyses based on data from one healthy individual [25]. However, no population-based longitudinal study or randomized controlled trial (RCT) has been published that can determine the bidirectional long-term causal relationships between sleep habits and brain structure.

Mendelian randomization (MR) analysis is an epidemiological study design that utilizes single nucleotide polymorphisms (SNPs) as instrumental variables (IVs) to establish causal effects of exposures on outcomes. This approach can be regarded as a natural RCT as it relies on the random allocation of alleles at gametogenesis, making it less prone to confounders and reverse causality compared to conventional observational multivariable regression. Previous studies have utilized MR analysis to explore the causal relationship between sleep and brain structure. However, these studies only examined the causal relationship between daytime napping and overall cortical volume [26], as well as sleep duration and overall cortical thickness [27]. A more comprehensive exploration of causal relationships between a wide range of sleep behaviors and global and regional brain structures is still warrant. This is critical for understanding what affects sleep habits and how adverse sleep behaviours contribute to a higher vulnerability to neuropsychiatric diseases. By elucidating the bidirectional causal relationships between sleep traits and brain structure, we may potentially provide guidance for prevention and intervention in psychiatric disorders and sleep disturbances.

Therefore, the objective of this study was to apply bidirectional two-sample MR analysis to investigate the causal effects between eight self-reported sleep phenotypes (namely insomnia complaint, sleep duration, long sleep, short sleep, chronotype, morningness, napping frequency and sleepiness severity) and 92 brain IDPs (including global and regional surface area [SA] and thickness [TH], volume of subcortical structures, and longitudinal changes in 15 brain structures) in adults of European ancestry, without any prior hypothesis.

## Results

### Overview of the study and instrument variants selection

**Fig. 1:**
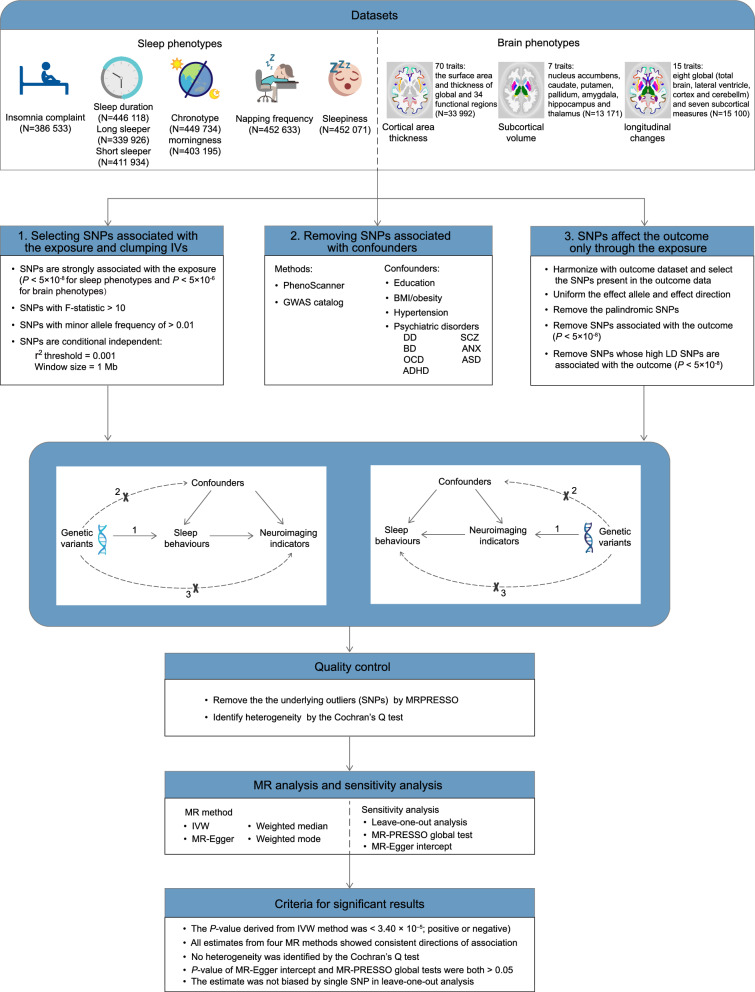
Flowchart of this bidirectional two-sample Mendelian randomization analysis. GWAS, genome-wide association studies; SNP, single-nucleotide polymorphism; BMI, body mass index; DD, depressive disorder; SCZ, schizophrenia; BD, bipolar disorder; ANX, anxiety disorder; OCD, obsessive–compulsive disorder; ASD, autism spectrum disorders; ADHD, attention deficit hyperactivity disorder; IVW, inverse-variance weighted; MR-PRESSO, MR Pleiotropy RESidual Sum and Outlier

**Fig. 2:**
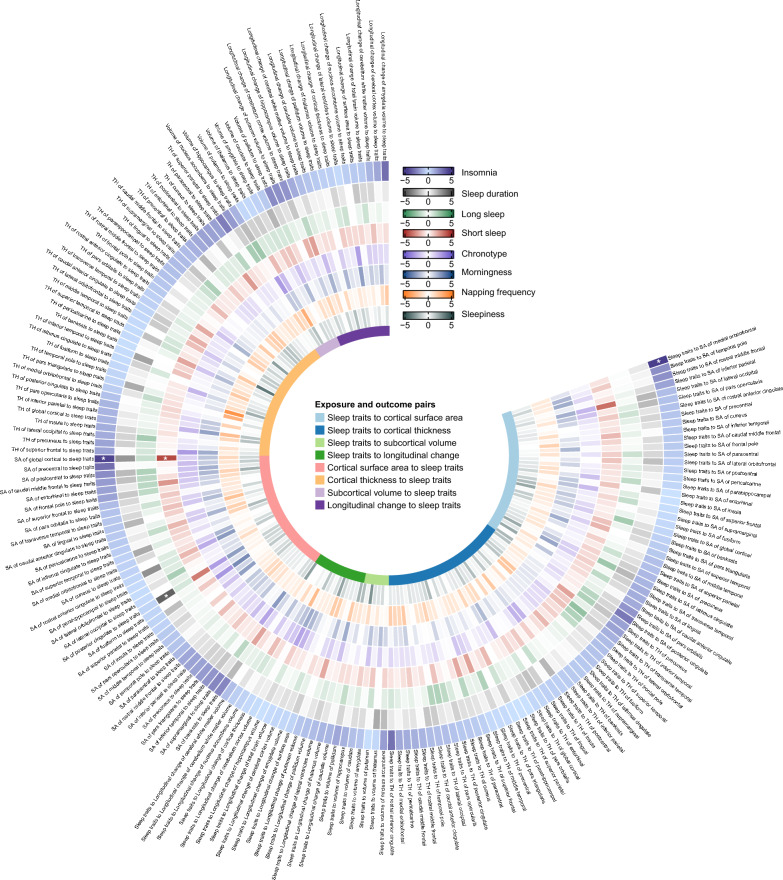
Z scores of all exposure-outcome pairs in the bidirectional two-sample Mendelian randomization analysis. All Z scores (β/SE) were derived using inverse-variance weighted method. The asterisk indicates significance after Bonferroni correction

We conducted a bidirectional two-sample MR analysis to investigate the relationships between all sleep traits-brain IDPs pairs. The study flowchart is presented in Fig. 1. To ensure that the samples used in the GWAS study for exposures were independent from those for the outcomes, we manually checked the sample description of each GWAS study. For subcortical brain structures, none of the participants were from the UK Biobank, indicating that there was no sample overlap between subcortical IDPs–sleep traits pairs; For cortical structures, 10 083 participants were from the UK Biobank, resulting in a sample overlap proportion of 29.7% (10,083/33,992); For longitudinal changes in brain structures, 2536 participants were from the UK Biobank, giving a sample overlap proportion of 16.8% (2536/15,100). After pruning for linkage disequilibrium, we removed the IVs associated with outcome as well as potential confounders. Outlier IVs detected by MR Pleiotropy RESidual Sum and Outlier (MR-PRESSO) outliers test were also excluded from exposure-related SNPs for subsequent MR analysis. The full lists of IVs used for forward and reverse MR tests were provided in Additional file 1: Table S1–S2. The Z scores of all exposure-outcome pairs derived from the random-effect inverse-variance weighted (IVW) method in the bidirectional two-sample MR analysis are showed in Fig. 2.

### Forward Mendelian randomization

**Fig. 3:**
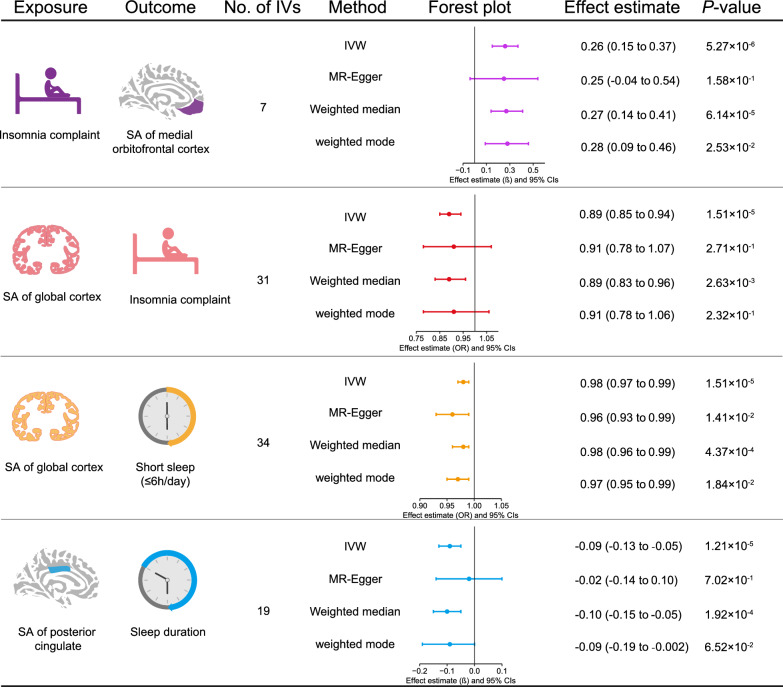
The significant causalities in the bidirectional two-sample Mendelian randomization analysis. These results all met the following criteria: the *P*-value derived from IVW method was < 3.40 × 10^–5^; all estimates from four Mendelian randomization methods showed consistent directions of association (positive or negative); no heterogeneity was identified by the Cochran’s Q test after removing the underlying outliers by MRPRESSO; *P*-value of MR-Egger intercept and MR-PRESSO global tests were both > 0.05; the estimate was not biased by single single-nucleotide polymorphism in leave-one-out analysis. SA, surface area; IVW, inverse-variance weighted; MR-PRESSO, MR Pleiotropy RESidual Sum and Outlier; IVs, instrumental variables

In the forward MR analysis, we identified that genetically predicted insomnia complaint was positively associated with the SA of medial orbitofrontal cortex (mOFC) (β, 0.26; 95% CI, 0.15–0.37; *P* = 5.27 × 10^–6^) (Fig. 3 and Additional file 1: Table S3). The other three MR methods corroborated this association (Fig. 3 and Additional file 1: Table S3). No heterogeneity was identified among the IVs for insomnia complaints based on the Cochran’s Q test (*P* = 0.899) (Additional file 1: Table S3). The *P*-values of MR-Egger intercept test and MR-PRESSO global test were 0.935 and 0.924, respectively, indicating the absence of horizontal pleiotropy (Additional file 1: Table S3). The scatter plot is shown in Additional file 1: Fig. S1. The estimate was not biased by any single SNP in the leave-one-out analysis (Additional file 1: Fig. S1). Furthermore, this association remained even when using non-overlapping IVs only in the MR analysis (β, 0.28; 95%CI 0.16–0.40; *P* = 3.65 × 10^–6^) (Additional file 1: Table S4). According to the study by Burgess et al. [28], the bias estimated from a 30% sample overlap for this exposure-outcome pair was less than 0.1%.

### Reverse Mendelian randomization

In the reverse MR analysis, we observed a negative correlation between the SA of global cortex and insomnia complaints (OR, 0.89; 95%CI, 0.85–0.94; *P* = 1.51 × 10^–5^), as well as short sleep (OR, 0.98; 95%CI, 0.97–0.99; *P* = 1.51 × 10^–5^) (Fig. 3 and Additional file 1: Table S5). We also found a negative association between the SA of posterior cingulate cortex (PCC) and sleep duration (β, − 0.09; 95%CI, − 0.13 to − 0.05; *P* = 1.21 × 10^–5^). The other three MR methods supported these associations (Fig. 3 and Additional file 1: Table S5). There was no heterogeneity or horizontal pleiotropy identified among these exposure-outcome pairs by the Cochran’s Q test, MR-Egger intercept test and MR-PRESSO global test (Additional file 1: Table S5). The scatter plots are shown in Additional file 1: Fig. S1, and the estimates were not biased by any single SNP in the leave-one-out analysis (Additional file 1: Fig. S1). There were no substantial alterations in the above associations when using non-overlapping SNPs only in the reverse MR analysis (Additional file 1: Table S6). According to the study by Burgess et al. [28], the bias estimated from a 30% sample overlap for these exposure-outcome pairs was all less than 0.1%.

## Discussion

In this study, we provided reliable evidence for the bidirectional causal relationships between a wide range of sleep habits and brain MRI morphological measures using summary-level data from large-scale GWAS. Forward MR analysis suggests that insomnia complaints have a causal effect on the SA of mOFC. Reverse MR analysis showed that individuals with reduced global cortical SA may be predisposed to experiencing insomnia complaints and short sleep (≤ 6 h/day), while reduced cortical SA in PCC could contribute to a vulnerability for longer sleep duration.

There is considerable interest in identifying modifiable risk factors that exert causal effects on brain structure because changes in brain structure can precede the onset of cognitive decline or dementia by several years. The finding that insomnia complaint was associated with the morphology of mOFC appears to be robust, given that previous sleep-related MRI literature have most frequently detected morphological alterations in the OFC or PFC not only among insomnia patients [10–13], but also in healthy individuals with higher insomnia severity [14], early-morning awakenings [15], and poor sleep quality [16, 18]. However, these studies were mostly cross-sectional, with the exception of one derived from a longitudinal cohort study in which sleep quality was assessed at only one time point [16]. Therefore, these studies could not determine whether alterations in grey matter (GM) morphology precede or follow the development of sleep disturbances.

Using genetic instruments identified in large-scale GWAS, this MR study identified robust evidence supporting a causal effect of insomnia complaints on the SA of mOFC. Considering that insufficient sleep has adverse effects on a variety of neurobiological processes that can potentially affect GM morphology (e.g., metabolite clearance and synaptic homeostasis), it is plausible that insomnia can directly impact brain structure. Some prior studies that identified a correlation between insomnia and morphological changes in the mOFC tended to support the view that sleep is a consequence rather than a contributor to mOFC structural abnormalities, as the GM reduction was not specifically related to insomnia duration [10, 11], contradicting the findings of this study. However, it is possible that insomnia duration may be nonlinearly correlated with OFC structural changes, with compensatory GM enlargement occurring during early stages followed by subsequent atrophy. If this hypothesis holds true, it would make sense that some studies observed GM hypertrophy in primary insomnia [12, 13, 17, 29], while others reported a negative correlation between insomnia and GM volume [10–12, 14]. Evidence supporting this speculation is that the mean insomnia duration of patients in the studies where cortical atrophy was observed were 7.6 and 17.7 years [10, 11], compared to 4.9 years in study where cortical hypertrophy was observed [13]. Future longitudinal studies are warranted to determine whether the neuropathology of insomnia is initial hypertrophy and later atrophy. Additionally, it cannot be denied that heterogeneity in sample size, demographic characteristics, neuroimaging analysis approach (e.g., surface-based morphometry and voxel-based morphometry) and brain imaging indicators (e.g., TH, volume and density) may also contribute to these inconsistencies.

The present study found a causal effect of insomnia on the SA in mOFC, specifically, insomnia complaint may lead to an increase rather than a decrease in it, which is consistent with a recent study reporting that primary insomnia patients showed cortical thickening and increased cortical volume in the left OFC [13]. Moreover, insomnia-related GM hypertrophy in other brain regions has also been reported [12, 17, 29]. These hypertrophic cortices can be explained by activity-related changes, for instance, skilled musicians showed increases in auditory cortical representation [30]; extensive learning tasks resulted in an increased volume of right hippocampal [31]. Therefore, the increased SA of mOFC maybe a compensation of persistent deleterious effect of hyperarousal, as the mOFC is involved in decision-making, behavioral flexibility, and social behavior. The cellular mechanism underlying the increased cortical SA is still unknown, potential process may include hypertrophy of neurons or glial cells, changes in the size or density of the capillaries, and remodeling of dendritic spines and synaptic connections [17]. Besides, shorter sleep duration and poorer sleep quality have been proved to be associated with greater Aβ burden [32]. Therefore, the increment of the sleep-related cortical SA may be explained by the space-occupying effects of amyloid plaques and other metabolic waste.

In addition, it is also possible that the relationship between insomnia complaints and SA of mOFC could be mediated by potential confounders. In the current study, the IVs used for final MR analysis excluded the SNPs associated with education, BMI/obesity, hypertension, and seven common psychiatric disease (DD, SCZ, BD, anxiety disorder [ANX], obsessive–compulsive disorder [OCD], autism spectrum disorders [ASD] and attention deficit hyperactivity disorder [ADHD]). Furthermore, age, sex, and a maximum of 10 principal components were included as confounding regressors in the primary GWAS studies.

Emerging evidence suggests complex causalities between sleep problems and neuropsychiatric disorders [3]. However, the mechanisms remain unclear. The causal relationship between insomnia complaints and SA alteration in mOFC, identified in this study, may provide insights into the underlying mechanism. The mOFC has functional connectivity with a number of brain regions involved in higher cognitive functions (such as hippocampus, amygdala, prefrontal lobe, dorsolateral thalamic nucleus, anterior cingulate gyrus, etc.) and plays an important role in the pathophysiological mechanisms of multiple psychiatric disorders (such as DD, SCZ, BD, OCD, ADHD, etc.) [33]. Sleep disturbances often precede the onset of psychiatric disorders, or develop in their early stages as the chief complaint on the first visit [34]. A study designed to describe residual symptoms in 943 patients with remission from major DD after treatment with citalopram reported that the most common residual symptom domains were sleep disturbance (71.7%), indicating that sleep disturbance (primarily insomnia) did not disappear with the remission of depression [35]. All these clinical phenomena suggest that insomnia may lie “upstream” in the causal chain of psychiatric disorders. Therefore, we propose the hypothesis that structural alterations in mOFC may partially mediate the link between insomnia and neuropsychiatric disorders, which needs to be tested in future studies. If the hypothesis holds, it is necessary to further explore whether sleep interventions can arrest, slow or reverse the progression of neuropsychiatric disorders. At the very least, our findings support that sleep patterns may be a cause of brain structural alteration, enhancing the importance of measuring and adjust for insomnia complaints in neuropsychiatric research aiming to delineate the morphological correlates or even antecedents of psychopathology. 

Alternatively, we also found that sleep architecture can be a consequence of brain structural abnormalities. In the reverse MR analysis, we identified a negative correlation of the SA of global cortex with insomnia complaint and short sleep, which may partially account for the higher prevalence of sleep impairment in older adults [36], since global brain atrophy is commonly seen as we age. Sleep problems are also frequently reported in neurodegenerative diseases [37], such as Alzheimer disease and Parkinson’s disease, the finding that brain atrophy could increase vulnerability to insomnia provides new insights into the mechanisms of developing chronic insomnia in neurodegenerative diseases and supports efforts to promote healthy brain aging through cognitive-behavioral therapy and lifestyle improvements. Interestingly, unlike the global cortical SA, the decreased SA in PCC may result in longer sleep duration. In the past few decades, scientists have devoted significant effort to identifying the subcortical brain regions responsible for wakefulness and sleep, and the possibility that cortical neurons regulate vigilance states has been overlooked. Recently, a study from Oxford University observed that silencing of layer 5 pyramidal and archicortical dentate gyrus granule cells in male mice markedly increased wakefulness and reduced rebound of slow-wave activity after sleep deprivation, supporting a role for the cortex in sleep homeostasis [38]. Additionally, the default mode network (DMN), a network of brain regions that display increased activity during wakeful rest in the absence of cognitively demanding tasks, plays a central role in the modulation of consciousness. During the transition from wakefulness to slow-wave sleep, functional connectivity within the DMN, particularly between the frontal (medial prefrontal, anterior cingulate) and posterior regions (PCC and precuneus) of the DMN, displayed a disconnected status, which is associated with the reduction in consciousness [39]. Therefore, it is plausible that a reduction in the SA of PCC may predispose individuals to sleep. However, the effect size was small, indicating that variables other than brain morphology significantly contribute to explaining the variance in sleep architecture.

There are some limitations in our study. First, sleep traits were self-reported rather than objectively measured, such as by using polysomnography, but it is usually not feasible in a large cohort study. Previous studies have suggested a moderate correlation between self-reported and objectively measured actigraphy [40]. Second, we did not explore the potential relationships between sleep habits and white matter microstructure, given that the GWAS data on human white matter microstructure were obtained from a meta-analysis of 43,802 subjects, including 36,624 from UK Biobank, which will lead to an overlap of up to 83.6% between the exposure and outcome samples. Third, in the inverse MR analysis, the genome-wide significance threshold was set at *P* < 5 × 10^−6^ due to the limited sample size of the brain structure GWAS, resulting in a lack of significant SNPs available when threshold was set at *P* < 5 × 10^−8^. This method of relaxing the statistical threshold for IVs has been used in previous high quality MR research when few associated SNPs are available [41]. Considering that this method carries the risk of introducing weak IVs, we selected SNPs with F statistics > 10 and conducted a series of sensitivity analyses. Only the consistent estimates from the four MR analyses, without horizontal pleiotropy and not biased by single SNP, were considered reliable. Fourth, there was partial overlap between the exposure and outcome datasets (29.7% for cortical structure and 16.8% for longitudinal change of brain structure), which may potentially bias the results. However, due to the lack of individual-level GWAS data, we were unable to remove the overlapping samples. Nonetheless, according to the study by Burgess et al. [28], the bias estimated from a 30% sample overlap was < 0.1% for the four causally associated exposure-outcome pairs identified in this MR research. Therefore, we speculate that the impact of sample overlap between GWAS studies of exposures and outcomes on our MR estimates is minor. Fifth, as the population we studied was Europeans, the estimates cannot be generalized to other races.

In conclusion, this study provided reliable evidence that sleep traits have a causal effect on brain GM structure. Additionally, it raised the possibility that the structural correlates of sleep measures in cross-sectional studies may represent pre-existing morphological GM deficits, which could increase susceptibility to certain sleep patterns. Undoubtedly, understanding whether modifiable lifestyle habits have a causal effect on the brain is crucial for allowing appropriate intervention. These findings imply that addressing and managing insomnia complaints may potentially mitigate changes in brain structural dynamics, thereby contributing to the prevention of neurological and psychiatric disorders. Further support for these suggestions is needed through additional sleep intervention trials and long-term cohort studies. This study also warrants further laboratory experiments to uncover the mechanism underlying the bidirectional causal relationships.

## Methods

### The aim, design and setting of the study

We conducted a bidirectional two-sample MR analysis to explore causal relationships between sleep behaviours and brain IDPs using summary-level results of published GWAS studies from the UK Biobank and the Enhancing Neuroimaging Genetics through Meta-Analysis (ENIGMA) Consortium. The study flowchart is presented in Fig. 1.

### The definition of sleep traits

1. Insomnia complaint: Participants were asked: “Do you have trouble falling asleep at night or do you wake up in the middle of the night?” with responses “Never/rarely,” “Sometimes,” and “Usually”. Participants who answered “usually” were considered as having insomnia complaints, while those who answered “never/rarely” or “sometimes” were defined as controls in the GWAS study [42].
2. Sleep duration, long and short sleep: Participants were asked: “About how many hours sleep do you get in every 24 h? (Please include naps)”, with responses in hour increments. Sleep duration was treated as a continuous variable in GWAS analysis. Binary variables for short sleep (≤ 6 h vs. 7–8 h) and long sleep (≥ 9 h vs. 7–8 h) were also derived. Extreme responses of less than 3 h or more than 18 h were excluded [43].
3. Chronotype and morningness: Participants were prompted to answer the question “Do you consider yourself to be?” with answers: “Definitely a ‘morning’ person”, “More a ‘morning’ than ‘evening’ person”, “More an ‘evening’ than a ‘morning’ person”, “Definitely an ‘evening’ person”, or “Do not know”, which were coded as 2, 1, − 1, − 2, and 0, respectively. Participants who answered “Definitely a ‘morning’ person” and “More a ‘morning’ than ‘evening’ person” were categorized as cases for morningness, while those who answered “Definitely an ‘evening’ person” and “More an ‘evening’ than a ‘morning’ person” were considered as controls [44].
4. Napping frequency: Participants were asked “Do you have a nap during the day?” with responses “Never/rarely”, “Sometimes”, “Usually”. The responses were treated as a continuous variable in the GWAS [45].
5. Daytime sleepiness: This phenotype was determined by asking the question “How likely are you to dose off or fall asleep during the daytime when you don’t mean to? (e.g., when working, reading or driving)” with the response options of “Never/rarely”, “sometimes”, “often”, and “all of the time”. The responses were coded continuously as one to four, corresponding to the severity of daytime sleepiness [46].

### GWAS summary data for sleep traits

Genetic variants associated with sleep traits, including insomnia complaints (N = 386,533) [42], sleep duration (N = 446,118) [43], long sleep (N = 339,926) [43], short sleep (N = 411,934) [43], chronotype (N = 449,734) [44], morningness (N = 403,195) [44], napping frequency (N = 452,633) [45], sleepiness severity (N = 452,071) [46], were available from corresponding GWAS studies conducted among European-ancestry adults in the UK Biobank. All associations have been adjusted for age, sex, a maximum of 10 principal components.

### GWAS summary data for brain imaging-derived phenotypes

The summary-level GWAS data correlated with the human cerebral cortex structure were obtained from a meta-analysis of the GWAS studies conducted among 33,992 European-ancestry participants, including 23,909 participants from 49 cohorts affiliated with the ENIGMA Consortium and 10,083 participants from the UK Biobank [47]. The cohorts that were enrolled are listed in Additional file 1: Table S7. The SA and TH of the whole cortex and 34 brain regions defined by the Desikan-Killiany atlas were extracted from structural brain MRI scans. The summary-level GWAS data correlated with the volumes of seven subcortical regions (nucleus accumbens, caudate, putamen, pallidum, amygdala, hippocampus and thalamus) were obtained from a meta-analysis of 13,171 individuals of European ancestry from 28 cohorts participating in the ENIGMA Consortium [48]. The cohorts that were enrolled are listed in Additional file 1: Table S8. The subcortical measures were extracted by the automatic subcortical segmentation software packages: FIRST, part of the FMRIB Software Library (FSL), and the FreeSurfer. The summary-level GWAS data correlated with the longitudinal changes in 15 brain structures across the human lifespan were obtained from a meta-analysis of 15,100 European-ancestry participants, including 12,564 participants from 35 cohorts affiliated with the ENIGMA Consortium and 2536 participants from the UK Biobank [49]. The cohorts that were enrolled are listed in Additional file 1: Table S9. The 15 brain structures consisted of seven subcortical structures and eight global brain measures (total brain including cerebellum and excluding brainstem, SA measured at the gray–white matter boundary, average cortical TH, total lateral ventricle volume and cortical and cerebellar gray and white matter volume). All of these measures were extracted using the FreeSurfer processing pipeline.

### Selection of instrument variant

We selected instrument variants (IVs) from two different GWAS summary results to perform a two-sample MR analysis, which can increase the estimated power. The selection of IVs should satisfy three assumptions: (1) IVs are strongly associated with exposure; (2) IVs should not be associated with potential confounders; (3) IVs influence the outcome only through the exposure of interest. To fulfill the first assumption, we chose two sets of *P*-values for genetic variants associated with the exposure in the bidirectional MR analysis. We used a threshold of *P* < 5 × 10^−8^ as the criterion for genome-wide significance to select IVs for estimating the causal effects of sleep traits on brain IDPs. In the inverse MR analysis for causal estimation of brain IDPs on sleep traits, the threshold of genome-wide significance was set at *P* < 5 × 10^−6^. We relaxed the statistical threshold for selecting IVs given that few (usually less than 4) or even none SNPs were identified when we used P < 5 × 10^−8^ as the threshold. However, as reported in the original GWAS study of the human brain structure [47], common variants explained 34% of the variation in total SA and 26% in average TH. These heritability estimates suggest that SNPs beyond those identified at *P* < 5 × 10^−8^ may contribute to variation in brain structure. This method of relaxing the statistical threshold for IVs has been used in previous high quality MR research when few associated SNPs are available [41]. Considering that this method carries the risk of introducing weak IVs, we calculated the F-statistic of each SNP and only the SNPs with F-statistic > 10 were retained. We also used linkage disequilibrium clumping (r^2^ > 0.001, and < 1 MB) to obtain independent SNPs associated with the exposure, and excluded the SNPs with minor allele frequency of < 0.01. For the second assumption, we removed SNPs associated with confounders that interfere with the association between brain structures and sleep traits. We determined age, sex, education, body mass index (BMI)/obesity, hypertension, and common psychiatric disorders (DD, SCZ, BD, ANX, OCD, ASD and ADHD) as confounders after reviewing a large amount of literature on the correlation between sleep and brain structure, as well as their respective influencing factors. These factors have been reported by previous studies to influence both sleep traits and brain IDPs [50–52]. In addition, these factors are also the most frequently adjusted covariates in sleep-related MRI studies [16, 53–56]. While some studies do not include mental status or psychological test scores as covariates, participants with psychiatric diseases are directly exclude [11, 20, 57]. Therefore, it is necessary for us to remove the SNPs related to these factors from the selected IVs for sleep traits or brain structures, in order to avoid attributing the causal effects of these factors to sleep habits or brain structure. Considering that all associations in the original GWAS studies have been adjusted for age, sex, a maximum of 10 principal components, then we further identified and removed the SNPs that are associated with education, BMI/obesity, hypertension, and common psychiatric disorders in the PhenoScanner database (http://www.phenoscanner.medschl.cam.ac.uk/) and the NHGRI-EBI GWAS catalog database (https://www.ebi.ac.uk/gwas/docs/file-downloads/) via PhenoScanner (version 1.0) [58] and gwasrapidd (version 0.99.13) [59] R Package. For the third assumption, we removed the outcome-related SNPs (p < 5 × 10^–8^) as well as the SNPs whose high LD SNPs are associated with the outcome. Besides, palindromic SNPs were also excluded after harmonizing the exposure and outcome data.

### Mendelian randomization analysis

Four different methods of MR (random-effect IVW [60], MR-Egger [61], weighted median [62], and weighted mode [63]) were conducted to obtain causal effects of the exposure on outcome, taking into account variants heterogeneity and pleiotropy effects. The random-effect IVW method was implemented as the primary statistical analysis because it provides estimates with the highest precision by combining the ratios of SNP-exposure to SNP-outcome in a random-effects meta-analysis to estimate the causal relationship between the exposure and outcome. However, this method relies on the assumption of no directional horizontal pleiotropy, as it constrains the intercept of the regression to zero. Therefore, MR-Egger, weighted median and weighted mode were performed to complement and enhance the robustness of the results. The MR-Egger approach models a pleiotropy parameter by fitting an intercept term, allowing it to detect and correct for directional pleiotropy, albeit with compromised power [61]. The weighted median method can yield valid causal effects if at least half of the weight in the analysis comes from valid IVs [62]. The weighted mode method provides consistent estimates when the relaxed IV assumption has less bias and a lower type I error rate [63]. When only one genetic instrument was available, we used the Wald ratio method for MR analysis.

### Sensitivity analysis

To improve the reliability of the genetic instruments, we conducted Cochran’s Q test to identify heterogeneity and removed any outlying SNPs by applying MR-PRESSO outliers test [64] before conducting the final MR analysis. We also performed the MR-Egger intercept test and MR-PRESSO global test to assess horizontal pleiotropy. Additionally, we applied leave-one-out analysis to check whether the causal relationship was mainly driven by a single SNP. To eliminate potential interference from overlapping IVs between exposure phenotypes, we re-conducted the MR analysis after removing the overlapping SNPs.

### Statistics

All analyses were performed using the TwoSampleMR package (version 0.5.6) in R (version 4.1.2). A Bonferroni-corrected *P*-value threshold was set at 3.40 × 10^−5^ (0.05/[92 × 8 × 2]; 92 represents the number of brain IDPs, eight represents the number of sleep traits, and two represents both forward and reverse MR analysis). The significant estimates should meet the following criteria: The *P*-value derived from IVW method was < 3.40 × 10^–5^; all estimates from the four MR methods showed consistent directions of association (either positive or negative); no heterogeneity was identified by the Cochran’s Q test after removing the outlying SNPs by MRPRESSO; the *P*-values of MR-Egger intercept and MR-PRESSO global tests were both > 0.05, indicating the absence of horizontal pleiotropy; the estimate was not biased by a single SNP, as indicated by the leave-one-out plot. In the forward MR, the effect estimates [β and 95% CI] for cortical structures were divided by standard deviation (SD) to visually measure the effect size. However, for subcortical structures and longitudinal changes of brain structures, the original results were presented given that the GWAS study did not provide SD information. In the reverse MR, the effect estimates for cortical structures were calculated as the change in sleep traits per SD change in brain IDPs, while the effect estimates for subcortical structures and longitudinal changes of brain structures were presented as original results. Moreover, for binary outcome, including short sleep, long sleep, and morningness, the effect estimates were transformed from [β and 95% CI] to [OR and 95% CI].

### Supplementary Information

**Additional file 1: Table S1.** Information of instrumental variables for all exposure-outcome pairs in forward Mendelian randomization analyses. **Table S2.** Information of instrumental variables for all exposure-outcome pairs in reverse Mendelian randomization analyses. **Table S3.** Forward Mendelian randomization analysis results. **Table S4.** Forward Mendelian randomization analysis results after removing the overlapping single nucleotide polymorphisms between exposure phenotypes. **Table S5.** Reverse Mendelian randomization analysis results. **Table S6.** Reverse Mendelian randomization analysis results after removing the overlapping single nucleotide polymorphisms between exposure phenotypes. **Table S7.** Descriptions of study cohorts participating in the genome-wide association studies meta-analysis to identify genetic variants associated with human cortical structure. **Table S8.** Descriptions of study cohorts participating in the genome-wide association studies meta-analysis to identify genetic variants associated with human subcortical brain structures. **Table S9.** Descriptions of study cohorts participating in the genome-wide association studies meta-analysis to identify genetic variants associated with longitudinal changes in brain structure across the lifespan.

**Additional file 2: Figure S1.** Scatter plots and leave-one-out plots of significant estimates in both forward and reverse Mendelian randomization analyses. SA, surficial area.

## Data Availability

The summary-level datasets of the human brain structure analyzed during the current study are available from the ENIGMA Consortium website, http://enigma.ini.usc.edu/research/download-enigma-gwas-results/. The datasets of the sleep traits analyzed during the current study are available from https://ctg.cncr.nl/ or http://sleepdisordergenetics.org/.

## Abbreviations

IDPs: Imaging-derived phenotypes
MR: Mendelian randomization
The ENIGMA Consortium: The Enhancing Neuroimaging Genetics through Meta-Analysis Consortium
SA: Surface area
TH: Thickness
MRI: Magnetic resonance imaging
RCT: Randomized controlled trial
SNPs: Single nucleotide polymorphisms
IVs: Instrumental variables
GWAS: Genome-wide association studies
FSL: FMRIB Software Library
BMI: Body mass index
DD: Depressive disorder
SCZ: Schizophrenia
BD: Bipolar disorder
ANX: Anxiety disorder
OCD: Obsessive–compulsive disorder
ASD: Autism spectrum disorders
ADHD: Attention deficit hyperactivity disorder
MR-PRESSO: MR Pleiotropy RESidual Sum and Outlier
IVW: Inverse-variance weighted
SD: Standard deviation
CI: Confidence interval
OR: Odds ratio
mOFC: Medial orbitofrontal cortex
PCC: Posterior cingulate cortex
PFC: Prefrontal cortex
DMN: Default mode network
GM: Grey matter
