# Supplementary material for: Causal associations between sleep traits and brain structure: a bidirectional Mendelian randomization study
Source: Behav Brain Funct. 2023 Oct 2;19:17. doi: 10.1186/s12993-023-00220-z (PMC10544625; doi:10.1186/s12993-023-00220-z)

**Supplementary Figure**

**Causal associations between sleep traits and brain structure: a bidirectional Mendelian randomization study**

Corresponding to Liankun Ren, Department of Neurology, Xuanwu Hospital, Capital Medical University, NO.45 Changchun Street, Xicheng District, Beijing, China. E-mail: renlk2022@outlook.com

**Figure S1** Scatter plots and leave-one-out plots of significant estimates in both forward and reverse Mendelian randomization analyses. SA, surficial area.


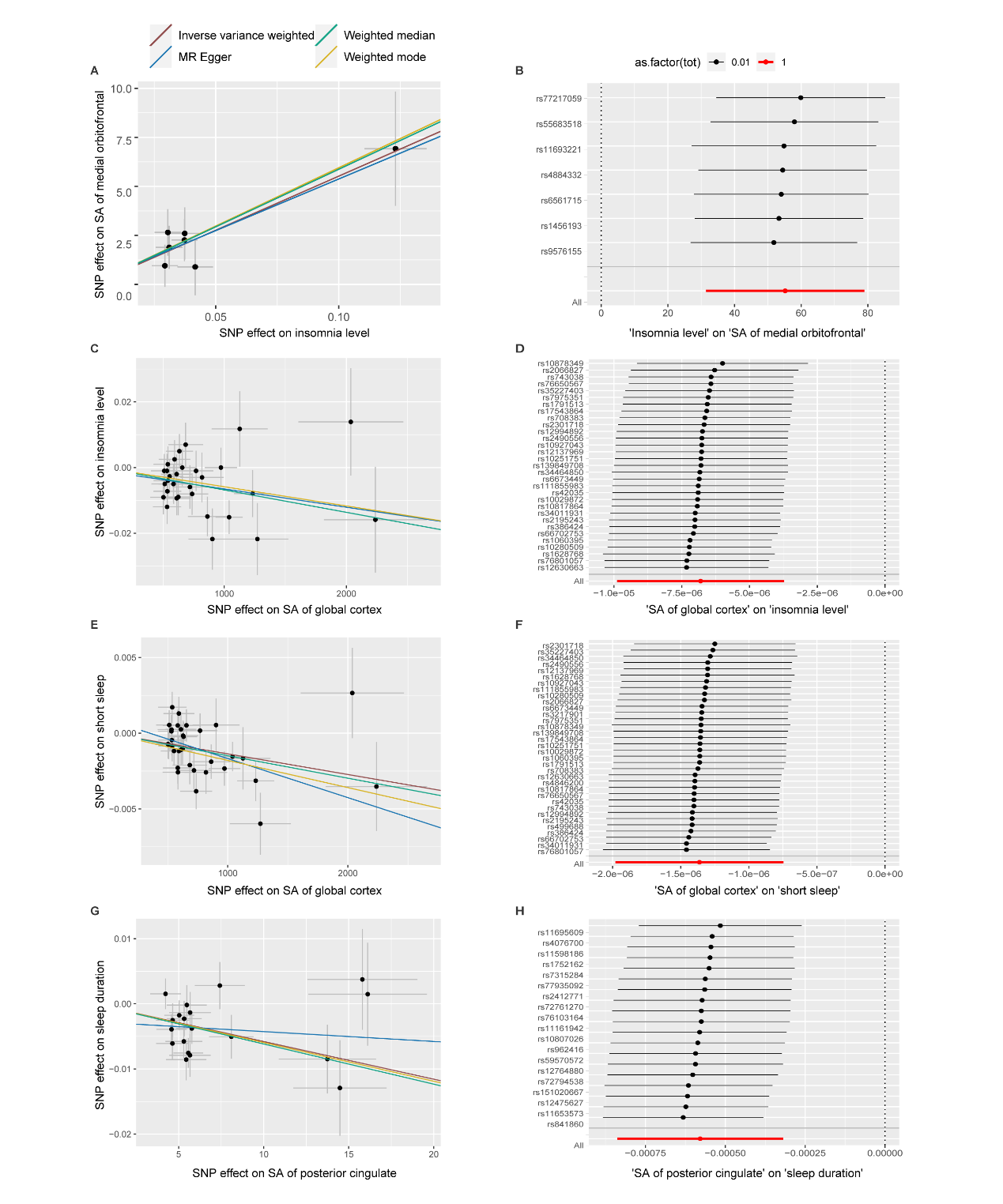

Supplement: Supplementary file 2 — Additional file 2: Figure S1. Scatter plots and leave-one-out plots of significant estimates in both forward and reverse Mendelian randomization analyses. SA, surficial area. [file 12993_2023_220_MOESM2_ESM.docx]
